# Radio and optical alignment method based on GPS

**DOI:** 10.1016/j.mex.2019.09.016

**Published:** 2019-09-17

**Authors:** M. Mariola, Y. Ismail, F. Petruccione

**Affiliations:** University of KwaZulu-Natal, Westville Campus, Durban, South Africa

**Keywords:** Coarse alignment for optical communication and fine alignment for radio communication, Tracking system, Optical transmission, Radio link

## Abstract

Point to point communication in free-space is severely dependent upon the alignment of the transmitter and receiver devices. The simplest low cost method for the alignment is achieved by utilising two geographical coordinates and an electronic compass. However, some regions of the Earth have a strong magnetic deviation that can introduce large errors to such systems. Other known methods, that can be utilised are a radio direction finder or stars sensor however these methods are too expensive. Here, we present a system which uses three GPS coordinates for the alignment of the transmitter and the receiver, of which two coordinates are measured on the transmitter side, while the receiver is previously known. The transmitter side positions can be relocated for convenience. The methods were tested using Google™ Maps for a long distance within the northern and southern hemisphere, while the experiment was performed for a short distance within the southern hemisphere. The system was developed based on the following considerations:

• Algorithm Implementable into a Micro-Controller Unit (MCU) or a standard computer.
• The local magnetic deviation does not have any influence on the method.
• Can be use where the internet connection is not available, such as mountains and others remote areas.

## Graphical abstract

**Figure:**
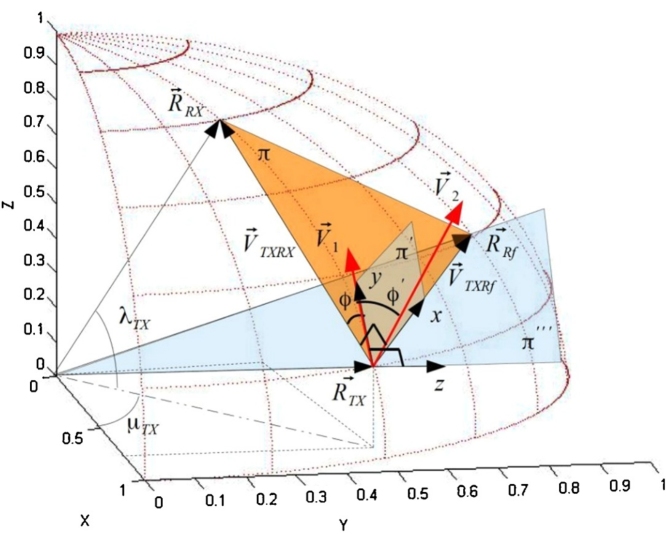

**Specification Table**

**Table:** 

Subject Area:	*Engineering and Physics*
More specific subject area:	*Free-space link communication*
Method name:	*Coarse alignment for optical communication and fine alignment for radio communication*
Name and reference of original method:	*Not applicable*
Resource availability:	*Laser 532 nm, GPS receiver, computer or Micro Controller Unit for authomated control, rotational stage (manual or authomatic)*

## Method details

**Fig. 1:**
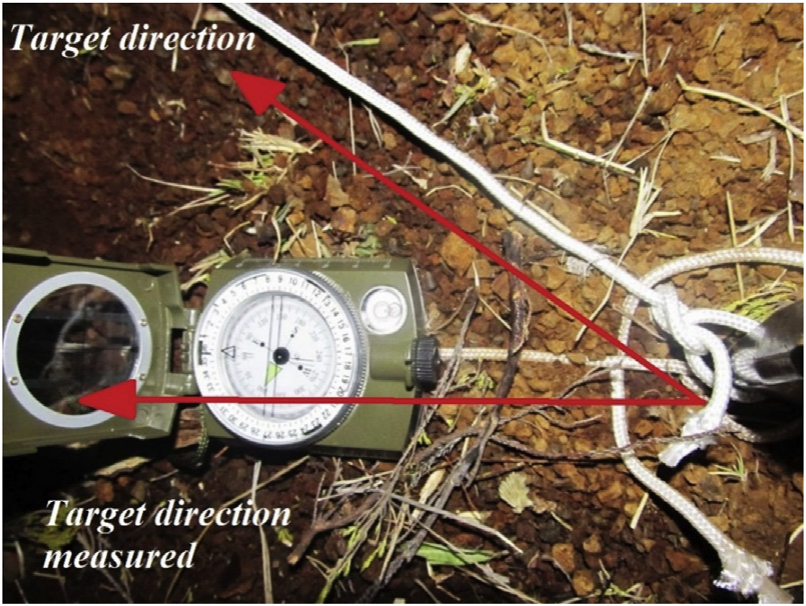
Magnetic deviation in Reunion Island. During an experiment, using a theodolite and a compass. The magnetic deviation in Piton Textor was greater than 20°.

Successful implementation of a free-space communication system operating at optical or radio frequency, is severely dependent upon the alignment of the transmitter and receiver devices. These devices can be an antenna or laser and an antenna or a photo detector respectively. Communication in free-space is required for civil applications such as the last miles and aerospace communications [1]. Optical communication in free-space is also implemented in quantum cryptography [2] and one of the requirements is an appropriate tracking system. To achieve the alignment of the transmitter and the receiver it is possible to subdivide the tracking system in two subsystems, that is, a coarse alignment system and a fine alignment system [3,4]. To date the coarse alignment is based on the Global Positioning System (GPS) coordinates of the transmitter and the receiver. Once the GPS coordinates are known, through an electronic compass, the transmitter is directed towards the receiver with a certain accuracy. Previous systems, according to literature, use the stars as a reference frame and the GPS coordinates of the transmitter and receiver to achieve the coarse alignment [5]. In some terrain there exist strong magnetic deviations due to geological factors which influences the alignment procedure as shown by the measured target direction in Fig. 1. Geographical coordinates, measured through the GPS hence can be affected by errors, which can be mitigated using the Differential GPS [6].

**Fig. 2:**
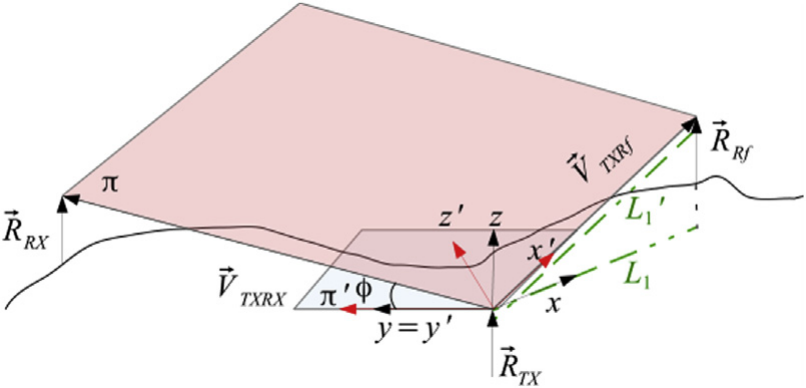
First alignment step: The initial direction of the laser $L_{1}$ is aligned with the x -axis which is the same direction of the maximum gain of the antenna or the laser used for the transmission. The z -axis is aligned with the direction of the position vector ${\vec{R}}_{\text{T}\text{X}}$ (position of the transmitter). In the first step the reference frame x, y and z is rotated around the y-axis until the x -axis is directed towards the position of the reference station. The new reference frame becomes $\;x^{'}$, $y^{'}$ and ${\;z}^{'}$.

The method proposed in this paper, uses three geographical coordinates, namely, the transmitter, receiver and a reference point called the reference station measured using a GPS. Each position is identified by the position vectors with respect to the origin on the center of the Earth. The position vector of the transmitter, receiver and the reference station are called $\;{\vec{R}}_{\text{T}\text{X}}$, ${\vec{R}}_{\text{R}\text{X}}$ and ${\vec{R}}_{\text{R}\text{f}}$ respectively. The transmitter, receiver and reference station can be operated by an end-user or be a stand-alone automated device. Initially the reference station and receiver coordinates are known by the transmitter. The standard Cartesian coordinates  x, y and z are used for the local reference frame of the transmitter. At first, the z -axis is aligned in the direction of the weight force parallel to the position vector ${\vec{R}}_{\text{T}\text{X}}$ as shown in Fig. 2 and the x -axis is aligned with a collimation laser ($L_{1}$) in the direction of the reference station located nearby the transmitter. The direction of the tracking laser $L_{1}$ is the same as the maximum gain direction of the antenna or the direction of the transmission laser used for the radio and optical communication respectively.

**Fig. 3:**
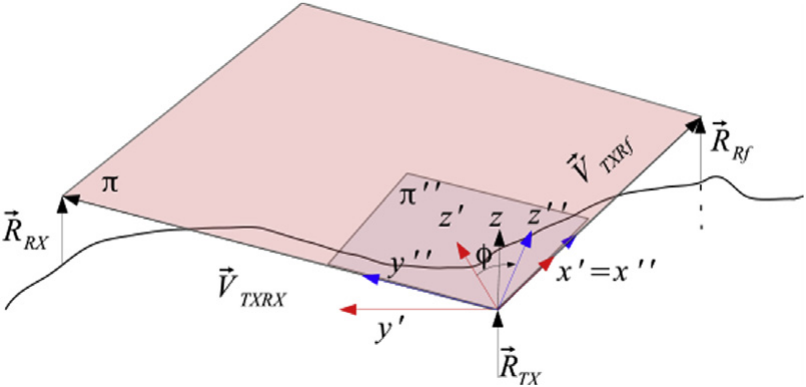
Second step followed by the algorithm. The new reference frame $x^{'}$, $y^{'}$ and $z^{'}$ is rotated by an angle ϕ in order to obtain a new reference frame $\;x^{\prime \prime }$, $y^{\prime \prime }$ and ${\;z}^{\prime \prime }$, where the plane $y^{\prime \prime }$ and $x^{\prime \prime }$ lie on the plane  π.

The procedure of the coarse alignment is based on undergoing a rotational transformation around the *y*-axis, the local reference frame translates to *x*′, *y*′ and *z*′ where *x*′ aims at the position of the reference station on the Earth surface. The plane where *x*′ and *y*′ lie is called *π*′ and is not coplanar with plane π identified by the positions ${\vec{R}}_{\text{T}\text{X}}$, ${\vec{R}}_{RX}$ and $\;{\vec{R}}_{\text{R}\text{f}}$. Through a rotation ϕ around the axis *x*′ the plane *π* and *π*′ becomes coplanar. The $z^{''}$ axis of the new reference frame *x*″, $y^{''}$ and $z^{''}$ is orthogonal with the plane *π*″, that is parallel to *π* as shown in Fig. 3.

**Fig. 4:**
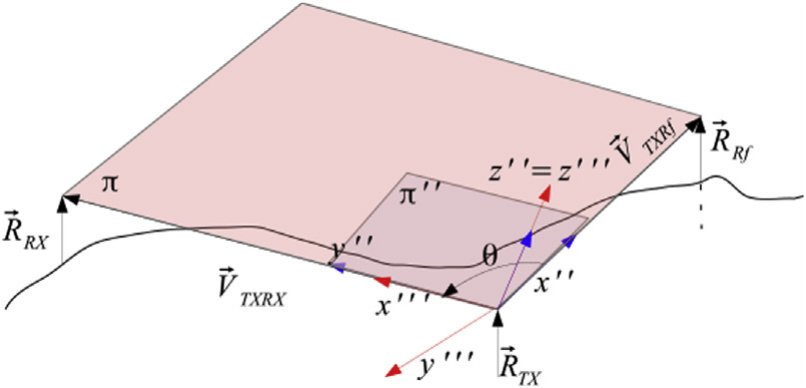
The last step involves the alignment of the transmitter to the receiver: After the previous rotation, the laser will aim in the direction of the reference station. By the rotation around $z^{\prime \prime }$ of an angle  θ, the transmitter is aligned with the receiver.

Another rotation of an angle θ around z'' ensures the alignment of the transmitter and the receiver as shown in Fig. 4.

## Three points GPS alignment

**Fig. 5:**
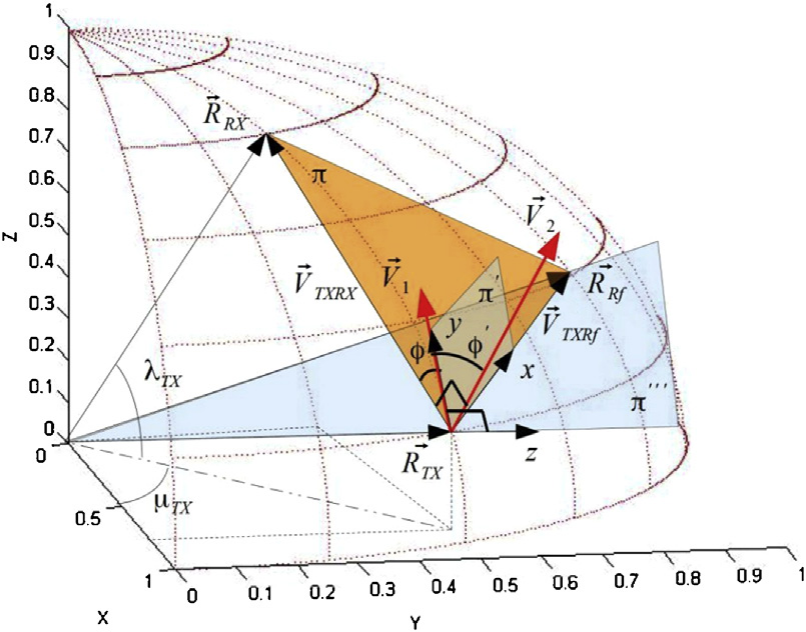
The planes and the relative angle used to calculate the directions of rotations. The position vectors are taken with respect to the centre of the Earth and are calculated by the WGS84 standard. By the GPS it is possible to measure the position on the Earth surface, the altitude from the geoid and from the ellipsoid.

To emphasis this method of coarse alignment, a fixed orthogonal reference frame  X, Y and Z which rotates in accordance to the rotation of the earth is chosen to identify the position vector ${\vec{R}}_{\text{T}\text{X}}$, ${\vec{R}}_{\text{R}\text{X}}$ and ${\vec{R}}_{\text{R}\text{f}}$. The origin of the reference frame is located on the center of the Earth where the X -axis has the direction of the Greenwich meridian, the Z -axis crossed through the geographical north pole and the Y -axis points in the direction 90° east with respect to the X -axis. This is shown in Fig. 5.

The Earth is not a perfect sphere and, to locate the position of the receiver, transmitter and the reference station, a standard WGS-84 was employed. This standard considers the Earth as an ellipse with the semi-major axis of a = 6378137 m and the semi-minor axis of b = 6356752.3142 m [7]. The altitude from the GPS is given from the ellipsoid of reference. The eccentricity e is expressed as:

(1) $$e^{2}=\frac{a^{2}-b^{2}}{a^{2}}=0.006694,$$

And the curvature of the first vertical is:

(2) $$N=N\left(\lambda \right)=\frac{a}{\sqrt{1-e^{2}{\text{sin}}^{2}\lambda }}$$

where λ is the latitude. The Cartesian coordinates of the position vector of the reference station are given by

(3) XRf=[N+hRf]cosλRfcosμRf,YRf=[N+hRf]cosλRfsinμRf,ZRf=[N(1-e2)+hRf]sinλRf. 

Here, $h_{\text{R}\text{f}}$ represents the altitude from the ellipsoid to the Earth’s surface of the reference station. The latitude and longitude are indicated by $\lambda_{\text{R}\text{f}}$ and $\mu_{\text{R}\text{f}}$ respectively. The position vector of the transmitter ${\vec{R}}_{TX}$ have the coordinates:

(4) XTX=[N+hTX]cosλTXcosμTXYTX=[N+hTX]cosλTXsinμTX,ZTX=[N(1-e2)+hTX]sinλTX, 

and the coordinates of the receiver are:

(5) XRX=[N+hRX]cosλRXcosμRX,YRX=[N+hRX]cosλRXsinμRX,ZRX=[N(1-e2)+hRX]sinλRX.

The subscripts  RX, TX and Rf represent the position of the receiver, transmitter and the reference station respectively. The vector ${\vec{R}}_{\text{T}\text{X}}$ is the position vector of the transmitter with respect to the chosen reference frame.

The angle θ, denoting the rotation of the transmitter in the direction of the receiver, is calculated by the inverse of the following inner product:

(6) $${\vec{V}}_{\text{T}\text{X}\text{R}\text{f}}.{\vec{V}}_{\text{T}\text{X}\text{R}\text{X}}=\left|{\vec{V}}_{\text{T}\text{X}\text{R}\text{f}}\right|\left|{\vec{V}}_{\text{T}\text{X}\text{R}\text{X}}\right|\text{cos}\text{θ}.$$

The direction of rotation (clockwise or anticlockwise) is calculated by the component Z of the cross product ${\vec{V}}_{3}={\vec{V}}_{\text{T}\text{X}\text{R}\text{f}}\times {\vec{V}}_{\text{T}\text{X}\text{R}\text{X}},$ where ${\vec{V}}_{\text{T}\text{X}\text{R}\text{f}}={\vec{R}}_{\text{R}\text{f}}-{\vec{R}}_{\text{T}\text{X}}$ and ${\vec{V}}_{\text{T}\text{X}\text{R}\text{X}}={\vec{R}}_{\text{R}\text{X}}-{\vec{R}}_{\text{T}\text{X}}$, as shown in Fig. 5. If the transmitter is located on the northern hemisphere and the component of ${\vec{V}}_{3}$ along Z is positive the direction of rotation is clockwise. If the transmitter is located on the southern hemisphere and the component of ${\vec{V}}_{3}$ along the Z -axis is negative, the direction of rotation is anticlockwise. Initially the direction of the laser $L_{1}$ is orthogonal to the vector ${\vec{R}}_{TX}$ and subsequently is tilted in order to be directed to the reference station. The local reference frame, y and z change to $x^{'}$, $y^{'}$ and $z^{'}$. The axis $x^{'}$ has the same direction of ${\vec{V}}_{\text{T}\text{X}\text{R}\text{f}}$ as shown in Fig. 5. For the transmitter, to be directed towards the receiver, it needs to be rotated around the vector ${\vec{V}}_{\text{T}\text{X}\text{R}\text{f}}$ once the angle θ is calculated. By the cross product, the vector ${\vec{V}}_{1}={\vec{R}}_{\text{T}\text{X}}\times {\vec{R}}_{\text{R}\text{f}}$ is orthogonal to the plane $\pi^{\prime \prime \prime }$ (see Fig. 5). Similarly, by the cross product the vector ${\vec{V}}_{2}={\vec{V}}_{\text{T}\text{X}\text{R}\text{f}}\times {\vec{V}}_{\text{T}\text{X}\text{R}\text{X}}$ is orthogonal to the plane. The angle $\varphi^{'}$ between the vectors ${\vec{V}}_{1}$ and ${\vec{V}}_{2}$ is calculated by the rules of the inner product. The plane $\pi^{\prime \prime \prime }$ is aligned with the plane given by x and z (see Fig. 2). The plane $\pi^{'}$ is coplanar with π when the vectors ${\vec{V}}_{1}$ and $\;{\vec{V}}_{2}$ are orthogonal. The angle of rotation around the vector ${\vec{V}}_{\text{T}\text{X}\text{R}\text{f}}$ is $\text{ϕ}=90^{\circ }-{\text{ϕ}}^{'}$. In the algorithm the component Z of the vector ${\vec{V}}_{1}$ must be the same as the component Z of the vector $\;{\vec{V}}_{2}$. During the calculation if the condition is false the cross product ${\vec{V}}_{2}$ becomes $\;{\vec{V}}_{2}={\vec{V}}_{\text{T}\text{X}\text{R}\text{X}}\times {\vec{V}}_{\text{T}\text{X}\text{R}\text{f}}$.

## Validation of the algorithm

The algorithm was tested by using the Google™ Maps software in order to measure the angle θ and compare the results calculated by using SciLab [8]. In the absence of infrastructure such as an internet connection or the presence of a strong magnetic field deviation the proposed method will adequately be able to coarse align a transmitter and receiver. As a proof of principle, an experiment was performed for a short distance in a soccer field, which is discussed in the next section.

For long distances using Google™ Maps the locations for the transmitter, receiver and the reference station were determined. The coordinates were converted in decimal degrees and by Eqs. (3), (4), (5) such that the components of the vectors were calculated. The simulation was done twice on the northern hemisphere and twice on the southern hemisphere. For the initial test the graphical software of Google™ Maps was used to measure the angle ${\text{θ}}_{\text{G}}$ between the direction of the transmitter-reference and transmitter-receiver stations. The angle between the vectors transmitter-reference station and transmitter-receiver $\theta_{\text{M}}$ was calculated using the double precision. Table 1 shows the algorithm for four testing conditions, and the error $\varepsilon_{\text{θ}\text{M}}=\left|{\text{θ}}_{\text{G}}-{\text{θ}}_{\text{M}}\right|$ between the measured and the calculated angle was performed.

**Table 1:** Verification of the algorithm in single precision using the Google™ maps software for four runs. The angles λ_X_, *μ*_X_, θ_X_ and ϕ_X_ are given in Degrees. The field D represents the direction of rotation between the direction of the transmitter-reference and transmitter-receiver stations. The time, t(μs) is the calculation time, necessary for the microcontroller to calculate the angle θ and φ.

	TEST (1)	TEST (2)	TEST (3)	TEST (4)
$\lambda_{\text{T}\text{X}}$	−29.06006944°	−32.60886667°	42.50960278°	41.43288333°
$\mu_{\text{T}\text{X}}$	27.24506667°	27.60504444°	11.56986389°	12.83350833°
$\lambda_{\text{R}\text{f}}$	−29.05515000°	−32.60844444°	42.50969167°	41.43427500°
$\mu_{\text{R}\text{f}}$	27.23995833°	27.60464444°	11.56978611°	12.83227778°
$\lambda_{\text{R}\text{X}}$	−29.00026667°	−32.60220833°	43.75004556°	41.48340278°
$\mu_{\text{R}\text{X}}$	27.30960833°	27.61673611°	12.24766389°	13.32911667°
${\text{θ}}_{\text{G}}$	85.6100°	94.1800°	54.1200°	115.6700°
${\text{θ}}_{\text{M}}$	85.8797°	94.7938°	54.5021°	115.7600°
*D*	Clockwise	Clockwise	Clockwise	Clockwise
${\text{ϕ}}_{M}$	0.0690°	0.1031°	0.9243°	0.3142°
$\varepsilon_{{\text{θ}}_{\text{M}}}$	0.2697°	0.6138°	0.3821°	0.09°
*d(km)*	9.1357	1.3227	148.43	41.7860

## Validation under real environmental conditions

In the absence of infrastructure, as mentioned previously, in remote areas the proposed method will be appropriate for the alignment of the transmitter and receiver. To validate this process a field test was implemented using a classical GPS, a laser lasing at 532 nm and a tripod with a protractor for civil constructions. The laser was fixed on top of the protractor and using a GPS the position of the transmitter TX was measured. A second GPS position was taken at the reference station $R_{\text{R}\text{f}}$ and at the position of the receiver RX. This experiment shows the feasibility of a coarse alignment using the GPS. The experiment was performed on the soccer field of the University of KwaZulu-Natal, Westville Campus (Durban, South Africa), over a distance of 18.91 m between the transmitter and the reference station. The distance of the link between transmitter and receiver was 75 m for TEST 1 and 93 m for TEST 2. Once the coordinates were acquired, the angle θ between the line of sight of the transmitter to reference station and the transmitter to receiver station was calculated by the proposed algorithm. The position of the reference station and the transmitter was fixed, while the receiver was relocated in order to test the algorithm for two different positions of the receiver. The alignment process was divided into four steps:

• Phase 1: Alignment of the transmitter $R_{\text{T}\text{X}}$ with the reference station $R_{\text{R}\text{f}}$
• Phase 2: Alignment of the laser with the reference station located on the goal post, 18.91 m away from the transmitter.
• Phase 3: Propagation of the laser in the calculated direction θ, where the receiver RX1 was located.
• Phase 4: Repetition of phase 3 for an alternative position of the receiver position RX2.

**Fig. 6:**
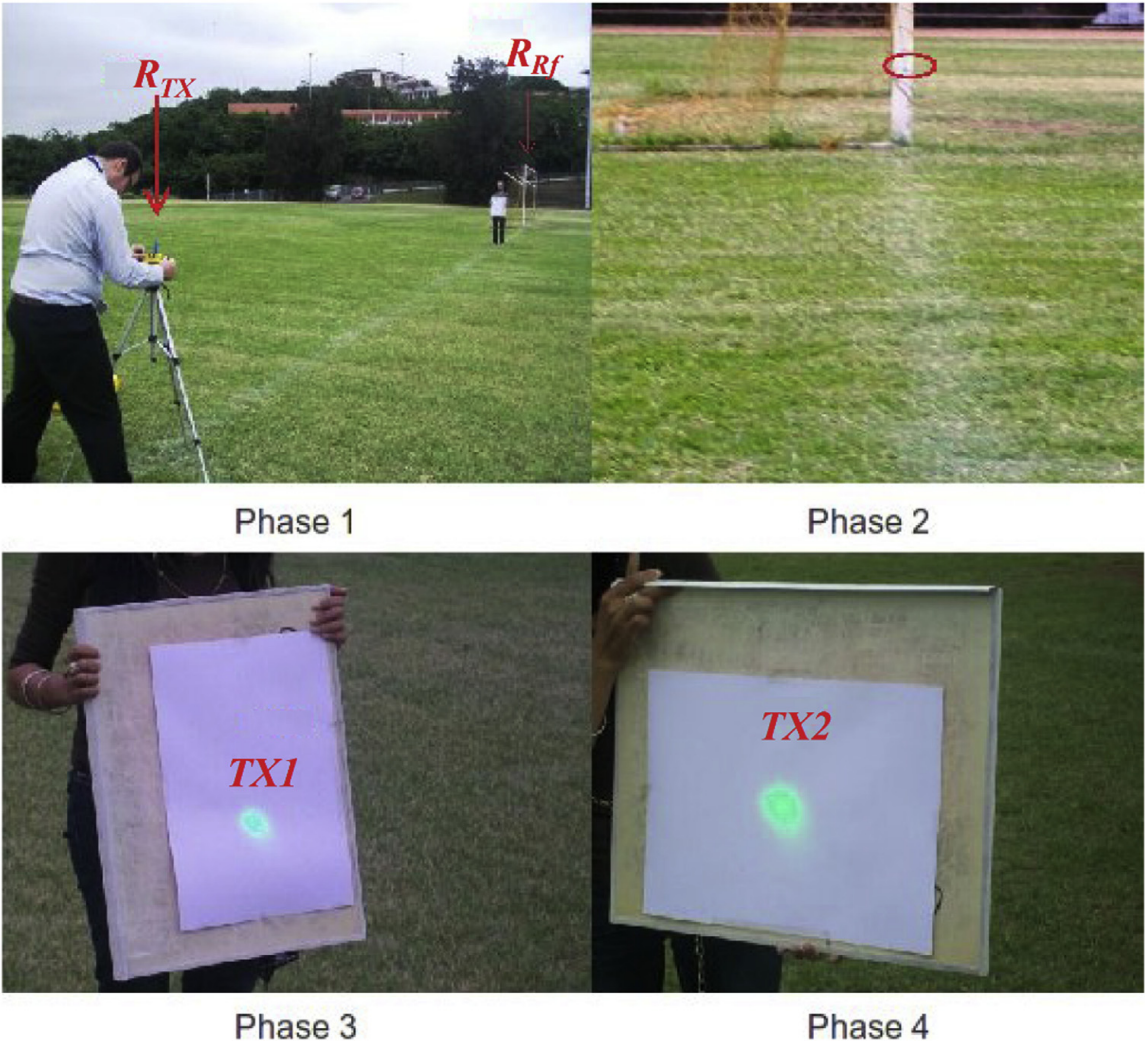
Experimental verification of the system performed for a short distance using double precision.

The results of the two measurements are shown in the Table 2 and graphically in Fig. 6 which is a further illustration of the accuracy of the system and the algorithm.

**Table 2:** Experimental verification of the algorithm in single precision at two positions of the receiver station along the soccer field.

	TEST (1)	TEST (2)
*λ*_TX_	−29.821365°	−29.821365°
*μ*_TX_	30.94651°	30.94651°
λ_Rf_	−29.821145°	−29.821145°
*μ*_Rf_	30.94636°	30.94636°
*λ*_RX_	−29.820775°	−29.82080375°
$\mu_{\text{R}\text{X}}$	30.946901667°	30.9472297917°
θ	60.798°	78.926°

## Conclusions

The error that occurs in the angle calculation can be neglected in several radio communication applications [9]. For optical communication the system can be used as a coarse alignment system, where more accuracy is required. With this method we were able to align the transmitter and the receiver station for short and long distances. The aforementioned method is expected to provide smaller errors for longer distances. Better performance can be achieved if a DGPS is utilised.
